# Can the integration of Motivational Interviewing skills in a virtual self-conversation be effective in promoting lifestyle changes among healthy adults and patients with obesity? A usability study

**DOI:** 10.1192/j.eurpsy.2022.1473

**Published:** 2022-09-01

**Authors:** D. Anastasiadou, J. Vázquez-De Sebastián, B. Spanlang, M. Slater, J.A. Quiroga, G. Parramón Puig, A. Ciudin, M. Comas, P. Lusilla-Palacios

**Affiliations:** 1 Vall d Hebron Institute of Research, Department Of Psychiatry, Group Of Psychiatry, Mental Health And Addiction, Barcelona, Spain; 2 Virtual Bodyworks S.L., Virtual Bodyworks S.l., Barcelona, Spain; 3 Vall d’Hebron University Hospital-VHIR Autonomous University of Barcelona, Department Of Psychiatry, Barcelona, Spain; 4 Vall d´Hebron University Hospital, Endocrinology And Nutrition Department, Barcelona, Spain

**Keywords:** Motivational Interview, obesity, embodiment, virtual reality

## Abstract

**Introduction:**

The integration of Motivational Interviewing (MI) with behavioural and psychological interventions for the treatment of obesity has the potential to improve health-related outcomes of patients in the long-term.

**Objectives:**

Our objective is to examine the usability of a VR embodiment tool for treating obesity.

**Methods:**

Fourteen participants (6 healthy and 8 with morbid obesity) with a desire to make lifestyle changes were randomly assigned to the experimental group (EG) and the control group (CG). Participants from the EG engaged in a virtual self-conversation aiming at understanding their own motivation to make lifestyle changes. Using the body swapping technique, participants were embodied alternately in their own virtual representation and in their counsellor’s body. To better guide this virtual self-conversation, participants were previously trained on MI skills. Participants from the CG were embodied in their own virtual bodies and participated in a “scripted dialogue” with a virtual counsellor who gave them practical recommendations about how to achieve lifestyle changes. A mixed-methods design was used, involving a semi-structured interview examining users´ satisfaction with the virtual experience, as well as self-report questionnaires, including readiness to change habits, body ownership, and system usability.

**Results:**

Participants showed high usability of the platform with higher scores among participants from the EG compared to the CG. Levels of body ownership were satisfactory, with no differences between groups.

**Conclusions:**

Through the integration of MI in the VR context with the patient being properly trained to carry out his/her own motivational self-conversation, we will provide an important advance in the psychological treatments of obesity.

**Disclosure:**

This project has received funding from the European Union’s Horizon 2020 research and innovation programme under grant agreement No 951930

